# Using biochemistry and biophysics to extinguish androgen receptor signaling in prostate cancer

**DOI:** 10.1074/jbc.REV120.012411

**Published:** 2021-01-09

**Authors:** Irfan Asangani, Ian A. Blair, Gregory Van Duyne, Vincent J. Hilser, Vera Moiseenkova-Bell, Stephen Plymate, Cynthia Sprenger, A. Joshua Wand, Trevor M. Penning

**Affiliations:** 1 Department Cancer Biology, Perelman School of Medicine University of Pennsylvania, Philadelphia, Pennsylvania, USA; 2 Department Systems Pharmacology & Translational Therapeutics, Perelman School of Medicine University of Pennsylvania, Philadelphia, Pennsylvania, USA; 3 Department of Biochemistry & Biophysics, Perelman School of Medicine University of Pennsylvania, Philadelphia, Pennsylvania, USA; 4 Department of Biology, Johns Hopkins University, Baltimore, Maryland, USA; 5 Division of Gerontology & Geriatric Medicine, Department of Medicine, University of Washington, and GRECC, Seattle, Washington, USA; 6 Department of Biochemistry & Biophysics, Texas A&M University, College Station, Texas, USA

**Keywords:** androgen receptor, allostery, cryo-electron microscopy, nuclear magnetic resonance, proteomics, mass spectrometry, aldo-keto reductase, splice variants, ADT, androgen deprivation therapy, AF, activation function, AKR1C3, aldo-keto reductase 1C3, AR, androgen receptor, ARSI, androgen receptor signaling inhibitor, CAK, CDK-activating kinase, CDK7, cyclin dependent kinase 7, CTC, circulating tumor cells, DBD, DNA-binding domain, DHT, 5α-dihydrotestosterone, EAM, ensemble allosteric model, FBDD, fragment-based drug discovery, HSP, heat shock protein, IHC, immunohistochemistry, LBD, ligand-binding domain, NR, nuclear receptors, NTD, N-terminal transactivation domain, qrtPCR, quantitative real-time polymerase chain reaction, PPI, protein–protein interaction, PSA, prostatic-specific antigen, RISH, rapid *in situ* hybridization, RM, reverse micelle, SHR, steroid hormone receptor, SID-IP-LC-MS, stable isotope dilution–immunoprecipitation–liquid chromatography–mass spectrometry, SILAC, stable isotope labeling of amino acids in cell culture, T, testosterone

## Abstract

Castration resistant prostate cancer (CRPC) continues to be androgen receptor (AR) driven. Inhibition of AR signaling in CRPC could be advanced using state-of-the-art biophysical and biochemical techniques. Structural characterization of AR and its complexes by cryo-electron microscopy would advance the development of N-terminal domain (NTD) and ligand-binding domain (LBD) antagonists. The structural basis of AR function is unlikely to be determined by any single structure due to the intrinsic disorder of its NTD, which not only interacts with coregulators but likely accounts for the constitutive activity of AR-splice variants (SV), which lack the LBD and emerge in CRPC. Using different AR constructs lacking the LBD, their effects on protein folding, DNA binding, and transcriptional activity could reveal how interdomain coupling explains the activity of AR-SVs. The AR also interacts with coregulators that promote chromatin looping. Elucidating the mechanisms involved can identify vulnerabilities to treat CRPC, which do not involve targeting the AR. Phosphorylation of the AR coactivator MED-1 by CDK7 is one mechanism that can be blocked by the use of CDK7 inhibitors. CRPC gains resistance to AR signaling inhibitors (ARSI). Drug resistance may involve AR-SVs, but their role requires their reliable quantification by SILAC–mass spectrometry during disease progression. ARSI drug resistance also occurs by intratumoral androgen biosynthesis catalyzed by AKR1C3 (type 5 17β-hydroxysteroid dehydrogenase), which is unique in that its acts as a coactivator of AR. Novel bifunctional inhibitors that competitively inhibit AKR1C3 and block its coactivator function could be developed using reverse-micelle NMR and fragment-based drug discovery.

Prostate cancer is a leading cause of cancer in the US male population resulting in 160,000 new cases per year and 30,000 deaths annually (1). Advanced prostate cancer can be treated with androgen deprivation therapy (ADT),^2^ which can include a surgical or chemical castration using the luteinizing hormone receptor agonist leuprolide and demonstrates that the disease has a reliance on androgen receptor (AR) signaling (2). Following a period of remission, the disease returns and is accompanied by the presence of a rising serum prostatic-specific antigen (PSA). PSA is an androgen-responsive gene raising the paradox of how this occurs in the presence of castrate levels of circulating testosterone (T). This paradox arises due to intratumoral androgen biosynthesis and changes in the AR itself. This form of the disease is known as castration resistant prostate cancer (CRPC) and is often fatal.

The AR is one of 48 human nuclear receptors (NRs) that function as transcriptional regulators, controlling a wide spectrum of processes involving metabolism, development, and reproduction (3, 4). The AR functions as a ligand-activated transcription factor and has a similar domain structure to other NRs. The N-terminal or transactivation domain (NTD) is followed by a DNA-binding domain (DBD), a hinge region, and a C-terminal ligand-binding domain (LBD). The AR is sequestered in the cytoplasm of target cells bound to heat shock proteins (HSPs). Binding of the potent androgens (T) and 5α-dihydrotestosterone (DHT), which is synthesized locally, leads to a reorganization of HSP interactions and exposes the nuclear translocation signal on the hinge region of the receptor resulting in subsequent translocation of the AR into the nucleus (5). The dimerized liganded receptor then binds to androgen-response elements (ARE) on ARE-driven genes to increase gene transcription, where each receptor binds to a half-site often arranged as inverted repeats. AR-driven genes lead to cellular proliferation and growth in prostate tumor cells, Figure 1.

**Figure 1:**
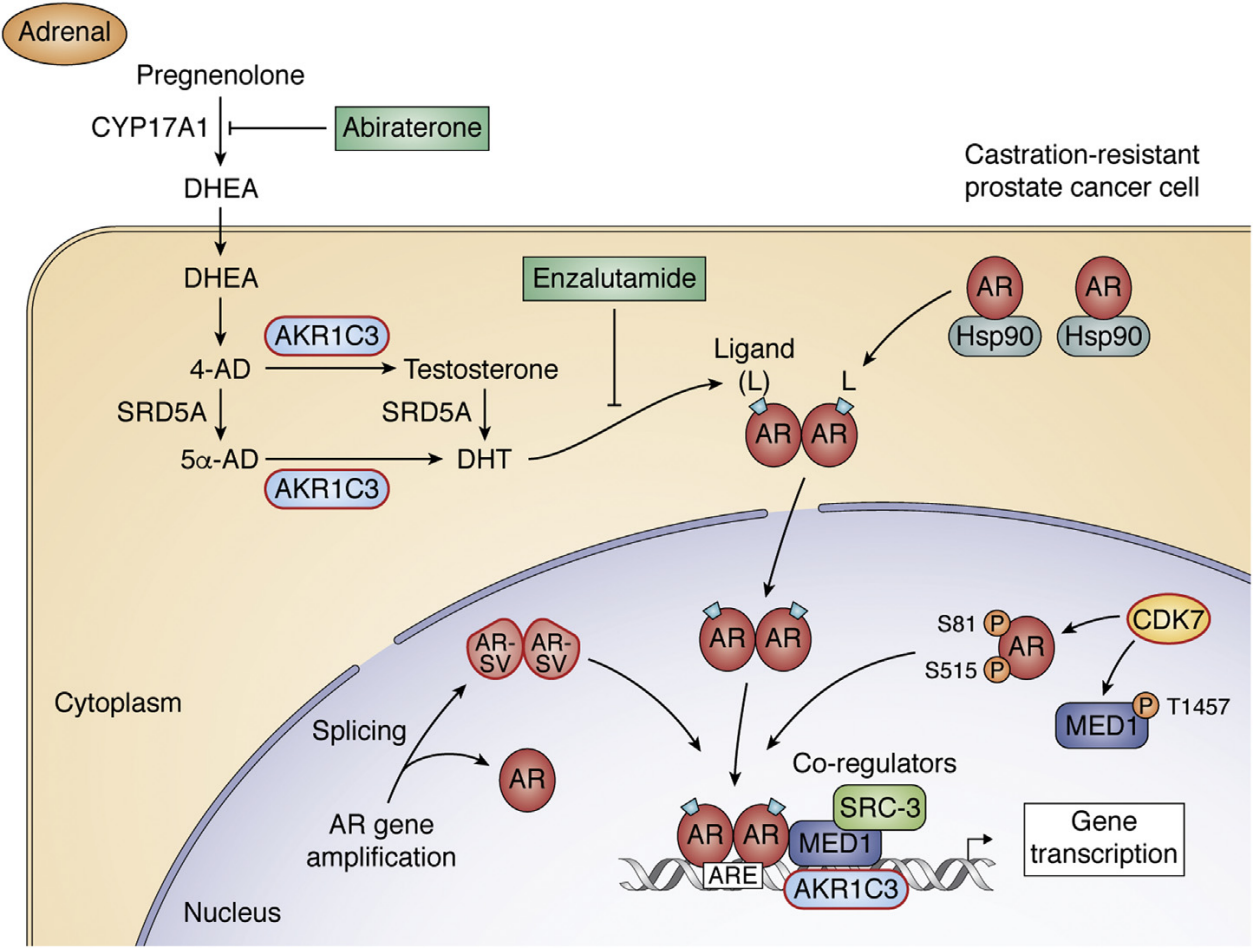
**Central role of AR signaling in prostate cancer and targets in CRPC.** Activation of the AR signaling pathway begins in CRPC with intratumoral testosterone (T) and dihydrotestosterone (DHT) synthesis catalyzed by AKR1C3. DHT then binds to AR sequestered by Hsp90 in the cytosol leading to translocation of the dimerized AR to the nucleus and its binding to androgen response elements (AREs) in the promoters of responsive genes. The sites of action of two ARSI therapies, abiraterone and enzalutamide, are shown. Alternative forms of the AR that are transcriptionally active in the absence of ligand are shown (AR-SV) and phosphorylated forms of AR. The recruitment of coregulators to the transcriptional complex is shown. Proteins in red boxes identify targets for the eradication of AR signaling. 4-AD, 4-androstene-3,17-dione; 5α-AD, 5α-androstane-3,17-dione; DHEA, dehydroepiandrosterone; L, ligand; P, phosphate; SRD5A, steroid 5α-reductase.

Standard of care for CRPC uses AR signaling inhibitor (ARSI) therapy to block steps in this pathway. The first class of agents includes the use of P450c17 (17α-hydroxylase-17/20 lyase) inhibitors such as abiraterone acetate (6, 7, 8) to inhibit the formation of adrenal dehydroepiandrosterone (DHEA) resulting in an inhibition of its conversion to potent androgens T and DHT in the tumor (intratumoral androgen biosynthesis). The second class of agents includes the use of the superior AR antagonists enzalutamide, apalutamide, and darolutamide (9, 10, 11, 12), Figure 1. However, the use of either type of agent results in ARSI drug resistance within 12 to 18 months and an increase in overall survival by only 3 to 4 months making these treatments ultimately ineffective (13, 14).

A number of mechanisms may be responsible for ARSI drug resistance, which are not mutually exclusive and some of the mechanisms are highlighted in Figure 1. These mechanisms include (a) adaptive intratumoral androgen biosynthesis (15); (b) AR gene amplification so that it can bind trace ligand (16); (c) mutation in LBD of the receptor so that it is ligand permissive (17, 18); (d) phosphorylation of AR so that it is constitutively active (19); and (e) appearance of AR splice variants (AR-SVs) that have lost their LBD and are now constitutively active (20, 21). The ability of different forms of the AR to activate gene transcription in the absence of ligand provides a mechanism to evade therapies that target the LBD. As CRPC remains dependent upon AR signaling (15, 22) there is widespread belief that the androgen axis must be extinguished to treat the disease, but this has yet to be accomplished.

More effective methods to block AR signaling are urgently needed, but the development of these approaches is hindered by significant knowledge gaps in how the AR functions. For example, the rational development of AR antagonists that target the LBD of the full-length receptor (AR-FL) or the NTD of AR and its variants is hindered by a lack of structural information that could be addressed by cryo-electron microscopy (cryo-EM) of AR and its complexes.

It is also clear that the domains of the AR do not work independently and that there is interdomain coupling that can be driven by the NTD, which is intrinsically disordered. The intrinsic disorder of the NTD needs to be probed using different domain constructs to elucidate how one domain affects the binding partners (coregulators, DNA, and small-molecule ligands) of another using the tools of fluorescence and CD spectrometry coupled with forced-folding techniques.

The AR also does not act alone but is further activated by coregulators and by phosphorylation to lead to transcriptional complexes on the chromatin. Elucidation of the mechanisms that lead to chromatin looping and enhanced gene transcription can lead to the identification of new therapeutic targets for the eradication of AR signaling.

The AR can evade ARSI therapy by the emergence of AR-SVs. The AR-SVs have been promoted as biomarkers of disease progression and ARSI drug resistance (23, 24). However, without a quantitative method to measure AR-FL and AR-SV by mass spectrometry, the prognostic and diagnostic value of AR-SV detection still remains uncertain. Stable isotope dilution–liquid chromatography–high-resolution mass spectrometry (SID-LC-HRMS) provides such a method.

ARSI drug resistance also occurs due to adaptive intratumoral androgen biosynthesis catalyzed by AKRC13 (type 5 17β-hydroxysteroid dehydrogenase) (15). AKR1C3 is one of the most highly upregulated steroidogenic genes in CRPC. A unique feature of AKR1C3 is its ability to act as a coactivator of AR. However, the allosteric mechanism that enables AKR1C3 to act as an AR coactivator is not understood. Bifunctional agents that simultaneously inhibit its enzyme and coactivator function could be better conceived if solution NMR structures of AKR1C3 complexes containing bifunctional agents were used to elaborate their mode of action. The design of superior bifunctional agents could be addressed by encapsulation and reverse micelle-NMR (RM-NMR) coupled with fragment-based drug discovery (FBDD) (see later). The advantages of these biochemical and biophysical approaches are listed in Table 1 and are elaborated in this review.

**Table 1:** Biophysical and biochemical approaches that can advance studies on AR signaling in prostate cancer

Biophysical/biochemical approach	Advantage	Application for AR signaling
Cryo-EM	• Macromolecular protein complex structures obtained at increasing resolution.	• Structures of AR-FL-ARE-coregulator complexes
	• Uses small amount of protein	• Structures of AR-SV-ARE complexes ± Coregulators
	• Does not require crystallization	• Structures of AR variant-ARE complexes with NTD or LBD antagonists
Fluorescence and forced folding using osmolytes	• Tryptophan fluorescence as a reporter of protein environment	• Use of two domain constructs (NTD) and (DBD) to determine their interaction
	• Fluorescence anisotropy to determine Kd values for binding partners	• Effects of two domains on DNA binding
	• Induced folding of intrinsic disordered domains	• Effects of two domains on transcription
Chromatin remodeling assays	• Determine role of different coregulator proteins in AR transcriptional complexes	• Coregulator antagonists
	• Determine mechanisms of coregulator recruitment	• Kinase inhibitors
		• Prevent phosphorylation of Mediator 1 complex and chromatin looping
Mass spectrometry	• SILAC labeling of internal standards	• Quantitation of AR-FL and AR-SV in prostate cells, PDX and patient biospecimens
	• LC-HRMS with parallel reaction monitoring used to quantify labeled and unlabeled peptides following in-gel digestion	• Determine whether ratios of AR-FL: AR-SV change upon disease progression
	• Quantitative *versus* qualitative immunochemical methods without concern for authenticity of Abs	• Determine whether AR-SV can predict ARSI drug response
NMR	• Solution structures permit elucidation of protein dynamics and ligand-induced allosterism	• AKR1C3 solution structure with bifunctional agents
	• Reverse-micelle (RM) encapsulation allows proteins to assume stable structures in confined space	• Scan AKR1C3 with NTD peptides to identify its coactivator domain
	• RM-FBDD allows screening of low-affinity binders to identify fragments that can be assembled into potent ligands	• FBDD to identify superior ligands for AR-NTD and AKR1C3

## Stopping AR function with antagonists

AR function could be stopped in its tracks with superior AR antagonists that target the LBD and prevent its binding to chromatin and by NTD antagonists that prevent its interaction with coregulators. Numerous structures have been reported for the AR LBD with agonist or antagonist bound (25, 26, 27, 28, 29). However, structures of the LBD with antagonist bound only exist for the mutated LBD where this domain now assumes a conformation in which the ligand acts as an agonist (26, 30). Thus no structure exists of the AR-LBD with antagonist bound in which AR is in the antagonist conformation impeding antagonist development. Only one structure has been reported for the DBD bound to an ARE (31).

**Figure 2:**
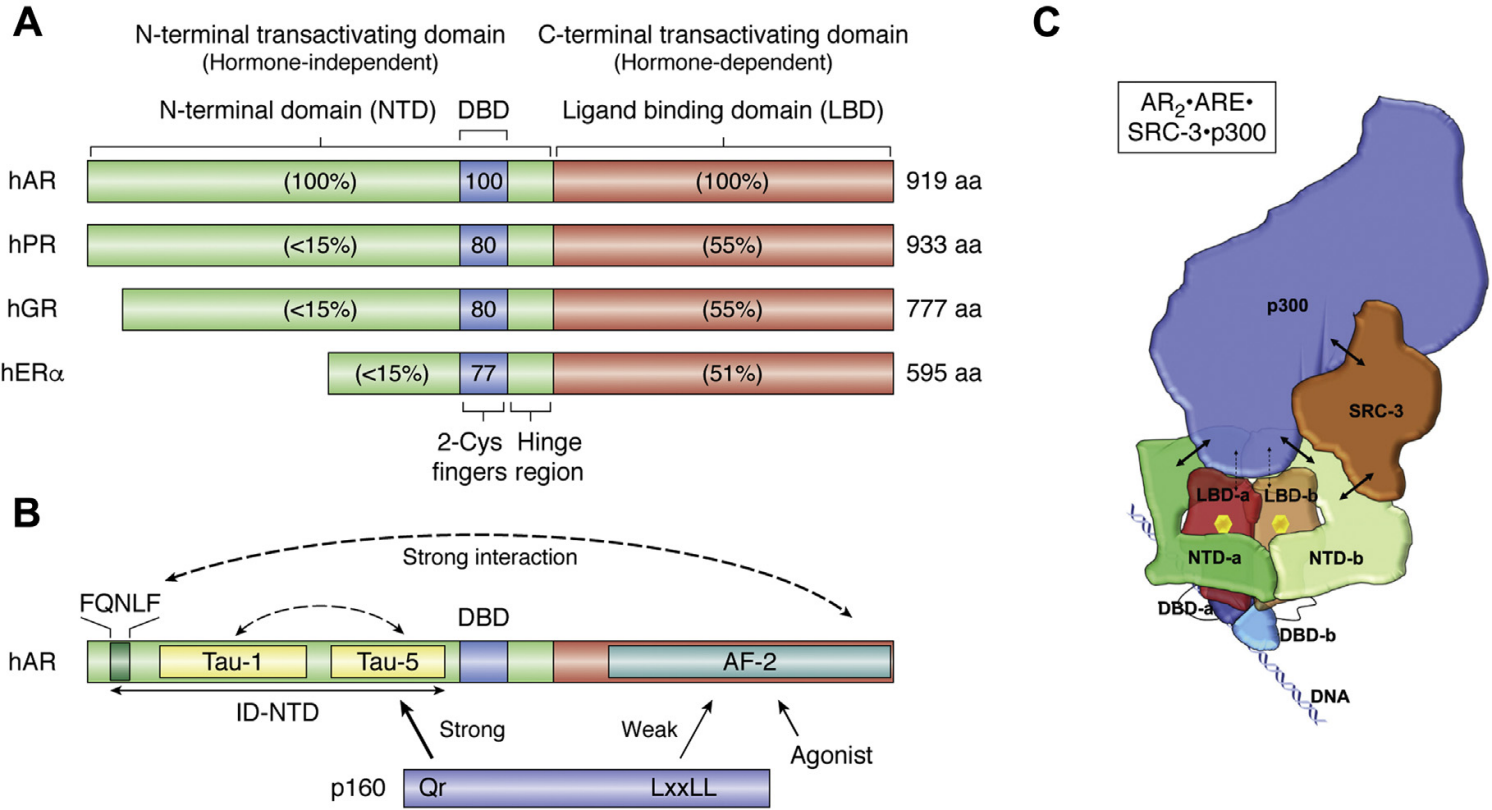
**AR Structural information.***A*, domain structure that is common to steroid hormone receptors: hAR, human androgen receptor; hPR, human progesterone receptor; hERα, human estrogen receptor alpha; hG, human glucocorticoid receptor. Numbers refer to the percent sequence identity in the domains where AR is 100%. *B*, inter- and intradomain interaction in hAR; the intrinsically disordered (ID) NTD contains the FQNLF motif and the AF1 region, which contains Tau-1 and Tau-5; DBD, DNA-binding domain and the LBD, ligand-binding domain also contains the AF2 region. The interaction with a p160 steroid receptor coactivator is shown; *C*, low-resolution cryo-EM of AR_2_•ARE•SRC-3•p300 containing R1881 obtained at 20 Å [from ref (37)].

The AR-LBD forms a bundle of 12 α-helices, where the central core of half of the domain makes up the androgen-binding pocket (28). The bound steroid is integral to the LBD structure and the unliganded domain is at least partially unfolded. Steroid binding generates a new binding pocket on the surface of the LBD termed activation function (AF)-2. In other steroid receptors, AF-2 binds LxxLL motifs found in coactivator proteins and is the key component of the coactivator recruitment mechanism (32). Coactivator proteins bind to steroid hormone receptors (SHR) and amplify transcriptional response in the presence of ligand (33).The AF-2 binding pocket in AR also binds to FxxLF motifs, including the FQNLF sequence found in the AR-NTD (34). Intramolecular FxxLF-LBD interactions therefore compete with coactivators that bind at AF-2 with LxxLL motifs (35, 36), Figure 2. In addition to modulating interactions with coregulators, the FxxLF-LBD interaction stabilizes androgen binding by slowing the steroid dissociation rate, effectively increasing the potency of agonists (34). This N-C interaction in AR is unique among the SHR family and is required for chromatin binding and for transcriptional activation at some promoters. Importantly, mutation of the LxxLL motif in SRC (steroid receptor coactivator)-3 showed that it was not required for interaction with AR, suggesting that the more important interaction of coregulators was with the NTD (37).

The AR-NTD recruits transcriptional coregulators, typified by members of the SRC/p160 family (4). The NTD also modulates the activity of the LBD, and the DBD, through a network of interdomain contacts. The NTD is intrinsically disordered and shares little sequence similarity with other NRs, confounding structural characterization of this region of the protein. The AR-NTD is among the largest in NRs (∼550 residues) and contains poly-glutamine and poly-glycine runs that vary among the human population (38), but have lengths of ∼23 residues each in the longest AR isoform; reference sequence (NCBI NP_000035). Most of the NTD is required for full constitutive and ligand-dependent transcriptional activation and contains two functionally independent activation regions found in its AF-1 domain referred to as Tau-1 (trans-activating unit 1) and Tau-5 (39), Figure 2.

The largest gap in our structural understanding of AR involves the NTD. It seems likely that at least some of the NTD will be structured in an AR-FL•ARE complex. Both the FQNLF motif and a related WHTLF motif in the NTD can interact with the LBD (34), and there is evidence from studies on the glucocorticoid receptor (GR) that residues in the NTD contact the DBD (40). Even more of the NTD should become ordered upon binding of coregulators, where the Tau-1 and Tau-5 regions are expected to mediate direct contacts. Given that coactivator recruitment is an essential component of the AR axis responsible for proliferation of prostate-derived tumors, understanding the structure and function of the AR-NTD is viewed as a high priority.

Several splice variants of the AR have been identified in patient-derived cancer cells and each of these lacks a functional LBD and their activity is driven by the NTD (41).The most common of these is AR-V7, which is truncated within the hinge region of AR. AR-V7 is a potent transcriptional activator that does not require androgens for activity, consistent with its frequent upregulation in tumors that have gained resistance to ARSI therapies (42). Whether AR-V7 signals as a homodimer or as a heterodimer with AR-FL and how this alters the transcriptome is under active investigation (43, 44, 45, 46).

### Biophysical approaches to the AR structure

Cryo-EM can provide information on large protein complexes, and this technology has been applied to the AR. In a recent landmark study, low-resolution cryo-EM structures of an AR_2_-ARE complex with R1881 (agonist) bound and of the same complex bound to its coregulators SRC-3 and p300 were reported at 13 Å and 20 Å resolution, respectively (37), Figure 2. These structures represented a major breakthrough and gave details of the gross architecture of these complexes without providing the high-resolution details to fully understand function. They also provide a path by which new AR complexes can be further interrogated by cryo-EM.

In the cryo-EM structures, AR dimerization was found to follow a unique head-to-head and tail-to-tail arrangement. The nonsymmetric nature of the AR_2_-ARE complex was revealed since only one NTD in the structure was identified by an AR-Ab that recognizes residues 39 to 97 suggesting that the two AR N/C interactions are not identical. Importantly, the NTD was found to be the primary site for coactivator binding but only one SRC-3 molecule was found in the AR_2_•ARE• SRC-3•p300 complex suggesting again that the two NTDs are not functionally equivalent in the dimer. It was proposed that the NTD-b recruits SRC-3 while NTD-a is involved in intra- and intermolecular N/C interactions. This structure differs from the cryo-EM structure of the ERα coactivator complex (47) where each monomer bound one molecule of SRC-3 and that the SRC-3 coregulators were bridged by p300, which did not contact ER itself (47). The recent AR_2_•ARE• SRC-3•p300 complex structure was built by merging single-particle cryo-EM images assuming that each particle represented the same structure. Because the AR domains allosterically regulate one another and predict that an ensemble of conformers is likely to exist for any single complex, merging particles assuming that they represent the same structure could be problematic and may provide an explanation for the low resolution of the structures thus far reported.

The structure of the AR_2_•ARE•SRC-3•p300 complex leaves many questions unanswered. It was hypothesized that the NTD exists in a number of conformations to account for how antagonists prevent coregulator recruitment to the NTD, that mutations in the LBD transmit information to the NTD so that antagonists now act as agonists, and that the NTD in AR-SV accounts for their transcriptional activity in the absence of the LBD (37). These hypotheses all support interdomain coupling within the AR but how and why this occurs is poorly understood from a structural perspective. The absence of a high-resolution structure for AR-FL and its complexes limits the development of antagonists that bind to either LBD or the NTD. NTD antagonists offer the promise of inhibiting signaling through AR-SV that remains constitutively active in the absence of the LBD (20, 21). One such compound in clinical trial is EPI-7836 but how it binds to the NTD is not known (48). Cryo-EM could also establish whether homodimers of AR-V7 or heterodimers of AR-FL and AR-SV bind to an ARE and whether this differentially regulates the formation of complexes with coregulators.

## Intrinsic disorder is the key to AR function

Many proteins are known to be intrinsically disordered (ID) or possess ID regions (IDRs) (49, 50, 51, 52, 53, 54, 55). Almost all transcription factors contain IDRs, and truncation or mutation of many such sequences significantly alters transcriptional activity (51). A general understanding of the mechanisms that underlie disorder-mediated allostery is a cornerstone to understanding not just how AR functions, but how all SHRs function in general.

IDRs sequences can adopt structure when they interact either specifically or promiscuously with binding partners (53, 56, 57, 58, 59, 60). The intrinsically disordered NTD in SHR serves an activation function and mediates allosteric communication between the various domains *via* several mechanisms. First, the addition of coregulators of GR, AR, and progesterone receptor (PR) induces secondary and tertiary structure within their respective AF-1 regions (58, 60, 61), suggesting that functionally important states exist within a disordered ensemble and that the ensemble can be redistributed in response to environmental cues. For AR, it appears that much of the NTD associated with its activation function interacts with one or a number of binding partners (58, 62, 63, 64, 65).

The second and most compelling feature is the ability of the DBD to induce structure in the NTD, indicating that structured and ID domains can communicate and even propagate information through each other (64). The fact that these effects were observed in different members of the SHR family suggests that, despite the low sequence conservation and significant differences in NTD length, there may nonetheless be a conserved mechanism of allosteric regulation.

**Figure 3:**
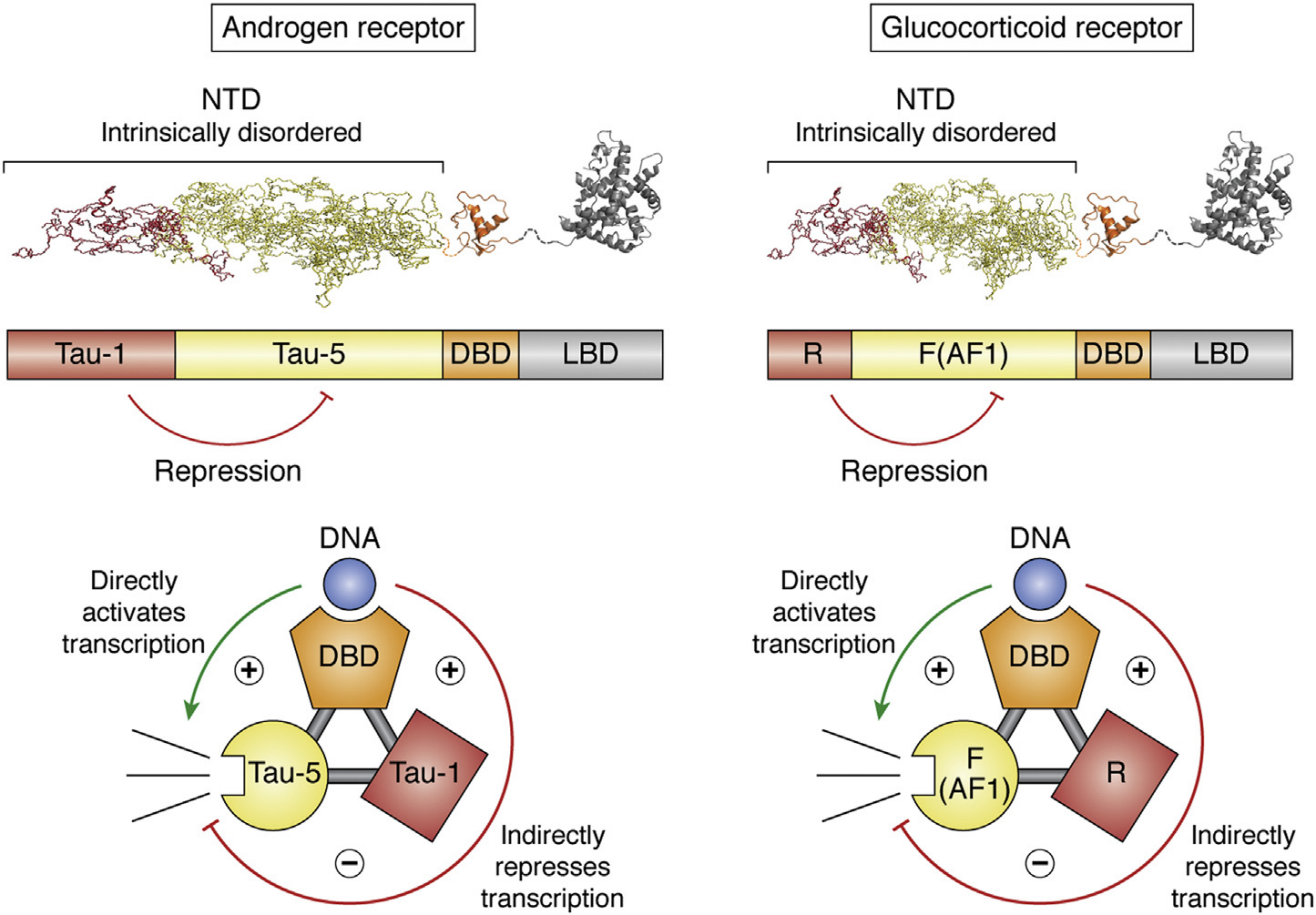
**Domain structure of androgen receptor (AR) and glucocorticoid receptor (GR) showing similar frustration in the NTD of each receptor.** For GR, the AF1-containing C-terminal portion of the NTD (*yellow*) is repressed by the N-terminal 98 a.a. (*red*), which serves as a regulatory element (R) (40). In AR, the NTD has two functional elements, TAU 1 (*red*) and TAU 5 (*yellow*). Like GR, the N-terminal portion of the NTD (*red*) represses the activity of the functional region (*yellow*). Unlike GR, where the regulatory region has no activity of its own, acting simply as a repressor, TAU 1 is both a repressor of TAU 5 and is responsible for ligand-dependent activation in AR by interacting with the LBD, (*Top*). Interdomain coupling where the DBD can activate transcription and the R domain leading to repression in GR similarly interdomain coupling where the DBD can activate transcription and the Tau-1 domain can lead to repression in AR (*bottom*).

Using the GR NTD as an example, a single AF-1 region is located centrally within the 425 amino acid NTD (61, 66). However, GR can be translated from either one of nine different conserved alternative translation start sites, six of which are located within the first 100 residues, producing different isoforms consisting of various lengths of the sequence preceding the intact AF-1 region (67, 68). Interestingly, these sequences negatively regulated (*i.e.*, repressed) GR activity and modulated DNA affinity in an isoform-specific manner (40, 67, 69). In other words, the NTD of GR can be viewed as being composed of two functionally distinct regions; 1) an AF-1 containing functional (or F) region that is responsible for transcriptional activation, and 2) an isoform-specific regulatory (or R) region that acts as a repressor of AF-1 function, with the different lengths of the R region for each isoform coding for different degrees of repression (40). Of note is that both the F and the R regions are stabilized by DNA binding to the DBD, leading to the discovery of a unique regulatory paradigm within GR, termed “thermodynamic frustration,” Figure 3 (70). In analogy to what will be described below for AR, the observed thermodynamic frustration in GR is potentiated by the existence of the negative (or repressing) interaction between the F and R regions of the NTD. These opposing effects can be quantitatively reconciled in the context of an ensemble allosteric model (EAM) in which the overall activity of GR (or any protein with such thermodynamic architecture (70, 71)) can be viewed as arising from contributions from two different subensembles (*i.e.*, an activating subensemble and a repressing subensemble), as shown schematically in Figure 3. The important feature of this model is that activation and repression are preencoded in the ensemble of states for all proteins (70, 71), and whether the overall effect of a perturbation (*i.e.*, binding, mutation, posttranslational modification, etc.) to a protein is either agonistic or antagonistic is determined by which subensemble is affected to the greatest degree. In the case of GR, this model demonstrates that DNA binding directly activates transcription, but at the same time indirectly represses transcription. The overall effect that is observed is based on the relative strengths of the activation and repression interactions, which vary for each isoform. The importance of this finding with regard to the SHR family is that it suggests that any ligand, coregulator, or even DNA may not obligatorily be either an activator or a repressor. To the contrary, the specific thermodynamic architecture of GR renders all molecules that bind GR to be “functionally pluripotent”(70, 71), meaning their effects have the potential to be manifested as an agonist or an antagonist, with the overall outcome being determined by which effect (*i.e.*, activation or repression) dominates, (see Fig. 3).

Can the results from GR be leveraged into an understanding of AR signaling? Clearly AR and GR share a common structural architecture, as do all of the SHR family (Fig. 2). However, the NTDs of GR and AR are also quite different, as their sequences reveal. Whereas GR has one AF-1 region, and 6 potential start sites upstream of the AF-1 region, the AR AF-1 region actually has two separable activation function regions; Tau-1, which mediates ligand-dependent activation, and Tau-5, which controls ligand-independent activation (72), Figures 2 and 3. AR, however, while not possessing alternative translation start sites upstream of the entire AF-1 region, as is the case for GR, does possess an alternative isoform (AR-A) that starts between the Tau-1 and Tau-5 site (73, 74), indicating that the shorter isoform (amino acid residues 189–919) possesses only the Tau-5, ligand-independent site. Furthermore, Claessens and coworkers have demonstrated that Tau-1 attenuates Tau-5 activity (75) suggesting that Tau-1, in addition to having its own activity, may serve as a regulator of the Tau-5 function in a manner similar to the way the GR AF-1 region is regulated by the upstream R domain. If so, this would suggest that AR and GR may have evolved different regulatory mechanisms that are built on a common thermodynamic architecture, an architecture that enables the effects of a particular ligand (*e.g.*, steroid, coregulator, or DNA) to be tuned by the environment for activation or repression. This EAM may help clarify the origins of the activation and repression differences in AR-FL and the AR-SVs and illuminate the molecular basis of ligand-independent signaling in CRPC.

### Biophysical approaches to interdomain coupling in AR

Biophysical approaches can be used to determine the basis of interdomain coupling in the AR. For example, using two domain constructs of the AR, which contain the NTD and DBD, permits the influence of one domain on the other to be studied in the absence of ligand. In these constructs the NTD can contain both Tau1 and Tau5 or only Tau5. Forced-folding experiments using the osmolyte trimethylamine oxide (TMAO) coupled with changes in intrinsic tryptophan fluorescence and CD spectroscopy can be used to determine the effect of NTD truncations on folding. As tryptophan residues become more buried, there is a blue shift in their emission spectrum. Knowing the maximal fluorescence change at any one concentration of TMAO, it is possible to determine the fraction of the construct that is fully folded. Using the same two domain constructs, the influence of NTD truncations on DNA binding to fluorescently labeled ARE half-sites can be measured by fluorescence anisotropy. Variable binding affinity to the ARE with different NTD constructs would illustrate allosteric modulation of DBD-binding affinity to the ARE. Functional consequences of this allosteric modulation can be measured in AR constructs in which the NTD is truncated in luciferase reporter gene assays in the presence and absence of ligand. Transitions observed in the TMAO and Ka associated with DNA binding give Gibbs free energies associated with them allowing a quantitative thermodynamic model to be established. Moreover, forced folding using the confinement of RM encapsulation of the different constructs followed by NMR allows structure of the ensemble of different folded states to be illuminated. Such an approach could determine how EP1-7836 acts as an NTD antagonist.

## The AR does not act alone

AR does not act alone but functions in a complex with other nuclear cofactors and chromatin-associated proteins to drive transcription of genes essential for tumor growth and drug resistance (76, 77, 78). There remains an impending need to better understand these complexes to develop effective treatments that can target AR and its associated proteins for drug refractory CRPC. Significant outstanding challenges related to AR signaling include the need: (i) to identify mediators of AR transcriptional signaling; (ii) to identify prognostic biomarkers that can discriminate between AR-driven CRPC and heterogeneous AR-independent neuroendocrine disease (see later); and (iii) to identify novel therapeutic targets in AR-addicted refractory CRPC.

Targeting of the proteins functionally associated with AR offers a promising new approach to block dysregulated AR signaling. Disruption of the AR interaction with coactivators such as the BET bromodomain family protein, the BRD4 methyltransferase complex, and the MLL-Menin, DNA repair enzymes—DNA-PKcs, have shown promising results in attenuating AR signaling and tumor growth in CRPC (76, 79, 80).

An important and critical coactivator of AR is MED1 (also known as TRAP220, PBP, and DRIP205), which forms a multiprotein Mediator complex that is required for enhancer-promoter looping and transcription pause release (81, 82). The Mediator complex functionally bridges DNA-bound SHRs such as AR and other mitogen-activated transcription factors with the basal transcriptional machinery composed of general transcription factors including TFIIA, TFIIB, TFIID, TFIIE, and TFIIH to facilitate the assembly and activation of RNA polymerase II (Pol II), which form the preinitiation complex at the core promoters (83). SHRs and the Mediator complex form phase-separated condensates at distal enhancers of highly expressed cell identity genes, which compartmentalize and concentrate the transcription machinery for rapid and copious transcription (84, 85). The Mediator complex is comprised of at least 30 subunits in humans that are arranged into four subcomplexes termed the head, middle, tail, and Cdk8-containing modules (81, 86). MED1 is the only subunit of this multiprotein complex that directly engages with ligand-activated SHRs and other cell type specific transcription factors that are essential for cell growth and development (84, 85). MED1 knockout in mice is embryonic lethal, and primary embryonic fibroblasts isolated from MED1 null embryos demonstrate retarded growth and cell cycle arrest (87, 88). Thus, in addition to interacting with nuclear receptors such as AR to act as a coactivator, MED1 may also play an essential role in cellular growth and cell cycle regulation. Consistent with this view, MED1 has been shown to bind the transcription factors involved in signaling, cell growth, and development, including the STAT family proteins, SMAD3, GATA family proteins, FOXA1, GABP (89, 90, 91, 92, 93).

MED1 binding to SHRs is mediated by LxxLL motifs in MED1 and the AF2 activation domain generated by ligand binding in SHRs (94). Additional studies have demonstrated the importance of the Tau 1 site in the NTD of AR for interaction with MED1 (95). MED1 is overexpressed in prostate cancer cells where it acts as an essential coactivator for AR-mediated transcription, and its knockdown results in cell-cycle arrest, decreased proliferation, and increased cell death (96, 97, 98, 99). Phosphorylation of MED1 at amino acid residues T1032 and T1457 by ERK, AKT, and DNA-PKcs promotes its association with other Mediator subunits and PolII, inducing chromatin looping by increasing recruitment of transcription factors and coactivators on chromatin (83, 93).

**Figure 4:**
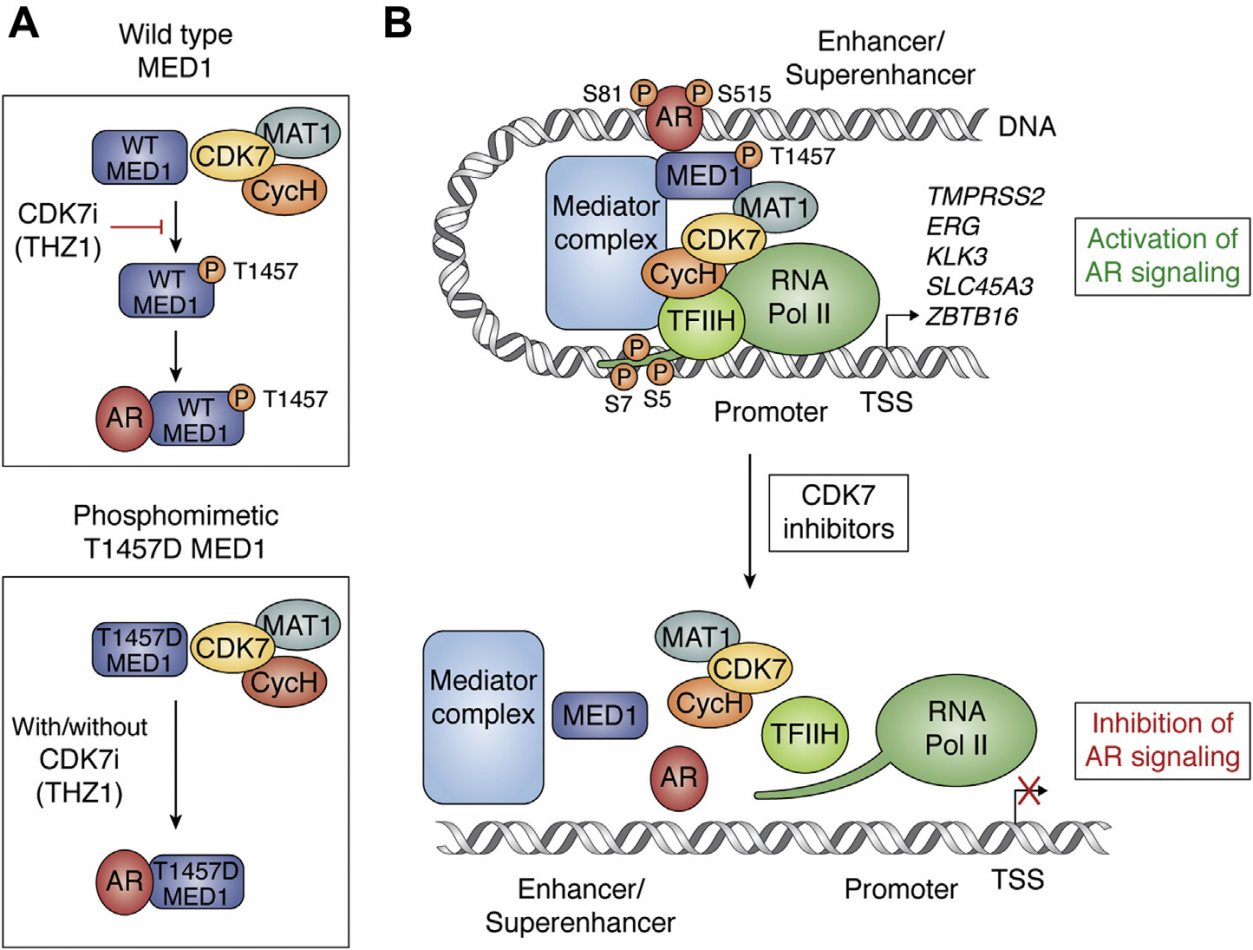
**CDK7 phosphorylation of MED1 at T1457 is essential for AR-MED1 interactions at the chromatin to active AR signaling**. *A*, CAK module of TFIIH basal transcription factor containing CDK7 phosphorylates MED1 at T1457, which is required for interaction with AR. CDK7 inhibition by small-molecule THZ1 disrupts MED1-AR interaction through the loss of pT1457. However, the phosphomimetic T1457D MED1 is constitutively bound to AR and is resistant to THZ1 treatment. *B*, potential mechanism of action of CDK7 inhibition in AR-addicted CRPC. AR binding to distal enhancer or superenhancer results in the recruitment of MED1 and other Mediator subunits, which then facilitates chromatin looping to the target promoter containing basal transcription factor TFIIH containing CDK7 and RNA Pol II. CDK7 then phosphorylates RNA Pol II at S5 and S7 of CTD heptapeptide and MED1 at T1457, which interacts with AR to form a stable AR-MED1 complex at the chromatin. CDK7 may also directly phosphorylate AR at S515 and S81, which may help stabilize the chromatin binding of AR. Treatment with CDK7 inhibitors leads to the collapse of the transcriptional complex at the AR target sites leading to loss of AR target gene expression

Studies have been performed to determine whether ligand-dependent AR activation leads to MED1 phosphorylation to promote its recruitment into the Mediator complex and interact with AR to form an active transcription complex (100). First, it was found that AR and MED1 were corecruited to chromatin in response to androgen stimulation and were enriched at enhancers and superenhancers. Inhibition of AR binding to chromatin by enzalutamide completely reversed MED1 binding, suggesting its recruitment is preceded by ligand-activated AR binding to AREs. Second, it was found that androgen stimulation increased MED1 phosphorylation at T1032 and T1457, while mutation to T1475A completely impaired its interaction with AR. Interestingly, the level of pT1457 was found to be associated with prostate cancer progression with metastatic CRPC tumors showing highest expression of this phosphorylated proteoform. Using a series of CDK inhibitors, reciprocal genetic knockdown, immunoprecipitation, and *in vitro* kinase assay, cyclin-dependent kinase 7 (CDK7) was identified as the direct kinase that targets T1457 for phosphorylation, while the T1457D phosphomimetic associates with AR on chromatin and is resistant to CDK7 inhibition (99) (Fig. 4*A*). Loss of CDK7 activity either through genetic inhibition or through treatment with THZ1, a small-molecule covalent inhibitor, leads to a loss of recruitment of MED1 to the chromatin, loss of AR-target gene expression, increased apoptosis, reduced cell proliferation, and inhibition of tumor growth in mouse xenograft CRPC models. Importantly, AR activity that was restored in enzalutamide-resistant prostate cancer cells was associated with increased MED1 pT1457 and demonstrated continued sensitivity to THZ1. The efficacy of THZ1 in curtailing hyper-AR signaling and promoting cell death even in the enzalutamide resistant setting points to the pivotal role of CDK7-MED1 axis in driving AR addiction and an approach to extinguish this activity (99) (Fig. 4*B*).

MED1 is also associated in the chromatin complex with phosphorylated AR (pS81 and pS515) where phosphorylation on S81 can lead to stabilization of the AR and constitutive activation of AR (19, 101) and phosphorylation of S515 mediated by EGF signaling can activate AR at reduced androgen levels (102). The kinases involved include CDKs, among others (*e.g.*, PKC for S81 and MAPK for S515) (102). It is also noteworthy that SRC-1, SRC-2, and SRC-3 share with MED1 the LxxLL coactivator peptide but likely mediate their action *via* the NTD. The SRCs contain histone acetyltransferase activity at the C terminus and can promote opening of the chromatin. In addition, SRC recruitment to the AR complex is facilitated by GATA2-binding protein paving the way for GATA2 small-molecule inhibitors such as K7174 to suppress the transcriptional function of AR (103). The SRC proteins are also targets for regulation by phosphorylation.

### Biochemical approaches—CDK inhibitors

CDK7, along with cyclin H and MAT1, comprises the CDK-activating kinase (CAK), which provides the T-loop phosphorylation required for activation of CDK1, CDK2, CDK4, and CDK6, which drive cell cycle progression (104). Additionally, the CAK module functions within the general transcription factor TFIIH to phosphorylate the Pol II heptapeptide carboxy-terminal domain repeats at S5 and S7, enabling promoter clearance and initiation (105). Due to its vital role in the cell cycle as well as transcription, CDK7 has emerged as a potential target for cancer therapy (106). Several CDK7-specific inhibitors have been developed that show efficacy against a wide range of cancer types, and their antitumor activity is likely mediated through cell cycle arrest and inhibition of oncogenic transcriptional addiction (107). Of note, three CDK7-specific inhibitors -CT7001 (Carrick Therapeutics-NCT03363893), LY3405105 (Eli Lilly and Company-NCT03770494), and SY-5609 (Syros Pharmaceuticals-NCT04247126) have entered phase 1/2 clinical trials for multiple malignancies (https://clinicaltrials.gov). However, only one of these trials includes CRPC patients.

In light of the preclinical data that supports a role of CDK7 to enhance AR activity, at least partly through phosphorylation of MED1, and increased MED1 phosphorylation contributes to the restoration of AR activity in enzalutamide refractory prostate cancer, CDK7 becomes a potential therapeutic target in advanced CRPC. Therefore, excitement exists in using CDK7-specific inhibitors in the clinic for advanced CRPC in which AR activity has been restored, where increased phospho-MED1 could serve as a biomarker to identify potential responders to such treatment.

## AR as an escape artist

AR may evade the effects of ARSIs by the appearance of AR-SVs that lack the LBD and are constitutively active. Examination of mRNA from patients in the international Stand Up 2 Cancer study demonstrated that 22 AR-SVs exist at the mRNA level (108). However, the role of each of these variants in prostate cancer progression is far from known. Currently, studies have identified the AR-V7 variant as the most prominent AR-SV. It is constitutively active, resistant to ARSI therapy, and could contribute to cancer progression (109).

AR-V7 transcripts are present in prostate cancer cells in the presence of androgen but are only detected at extremely high PCR cycle numbers and no AR-V7 protein is seen on western blot (110). Subsequently rapid *in situ* hybridization (RISH) also showed low levels of mRNA in primary tumors without concomitant protein expression (111). The role that these low levels of mRNA may play in the absence of dectable protein expression is unknown. Data show that the mechanisms for aberrant splicing of the AR can be rapidly increased to produce AR-V7 in the presence of androgen deprivation. Importantly, AR-V7 protein is rarely, if ever, found in primary prostate cancer specimens, appearing only after ADT and ARSI therapy (112, 113). In castration-sensitive prostate cancer, the incidence of AR-V7 protein expression in metastatic tissue, as measured by IHC, is <0.1% of tumors, but this increases to a 75% incidence in ADT-resistant prostate cancer and >85% in abiraterone and enzalutamide-resistant prostate cancer. In addition, the number of tumor cells expressing AR-V7 and the intensity of AR-V7 expression are significantly higher after ARSI therapy, *p* < 0.02 (113). Reanalysis of studies purported to demonstrate AR-V7 in primary prostate cancers (114) shows that 23% of the reported primary cases had actually received neoadjuvant ADT for a mean of 35 days prior to obtaining prostatectomy samples. This exposure to ADT then would account for the 19 positive AR-V7 primary cases in this study. Also, in castrate-sensitive prostate cancer patients, who showed a significant percentage of AR-V7 positivity, an antibody dilution of 1:50 was used and demonstrated significant cytoplasmic staining, which is inconsistent with the nuclear location of AR-V7 (114).

In 2014, Antonarakis, *et al.*, (23) measured AR-V7 mRNA levels in circulating tumor cells (CTCs) and demonstrated that the presence of AR-V7 could be used as a marker of resistance to ARSI therapies. Since then, multiple groups have examined the correlation between the presence of AR-V7 mRNA in CTCs and therapeutic drug resistance (23). It has been suggested that the detection of AR-V7 protein in CTCs by AR-V7-specific antibodies is not only a marker of drug resistance but also can be used to determine choice of treatment, whereby ARSI therapy is replaced with taxane therapy (24, 115, 116). However, several issues concerning this approach must be considered. First, the readout on either mRNA in CTCs or AR-V7 protein is a binary measurement (117). Second, intrapatient tumor heterogeneity with contemporaneous AR-V7-positive and AR-V7-negative CTCs is an issue. Third, it is not known how such variation is reflected in CTCs from the same patients. Fourth, CTC studies must also consider the fact that many patients with CRPC, especially if they are chemotherapy naive, have few to no CTCs in their blood upon which to perform such measurements. Fifth, there is no reliable method to measure the protein mass of AR-FL or AR-SV. Finally, studies must correlate response to treatment with assay positivity and not just survival data. Circulating CTC measurements may one day develop into an important and useful assay based on AR-V7 measurements, but further nonbinary and specific quantitative protein assays must first be developed.

There are an array of techniques currently in use to detect the presence of AR-SV mRNA and protein levels in CTCs as well as in whole tissues (109, 118). Early studies using qrtPCR suggested that the amount of AR-SV mRNA compared with levels of AR-FL was less than 1% (119). More recent examination of mRNA in tumor samples, using RISH with probes specific for the exon 3/cryptic exon 4 junction found in AR-V7, suggests that the amount of AR-V7 mRNA in tissue samples is greater than 1% (118). Examination of protein expression is now possible with the development of AR-V7-specific antibodies. Like the RISH studies, studies examining AR-V7 protein levels suggest that it is present at higher levels than suggested by qrtPCR (118). Importantly, RISH and IHC studies have also demonstrated that there is heterogeneity in AR-V7 expression both within in a single tumor sample and across metastatic samples from the same patient (113). Equally important is the finding that men may have negative CTC AR-V7 tests but show significant areas of AR-V7 staining in their tumors and vice versa. This discordance could be due to how researchers define a positive cutoff level in qrtPCR as well as the use of different PCR primers between groups. In addition, there is the possibility of false-positive staining with AR-V7-specific antibodies (113). Semiquantitative studies of the AR in prostate tissue and CTC currently rely on the determination of mRNA levels (120, 121), or immunohistochemical analysis of AR-SVs (122) These studies reveal variable expression of the AR variant mRNA that arises by skipping of exons 5, 6, and 7 (AR^v567es^ or AR-V12); it is highly doubtful that these data accurately reflect the role of the AR-SV in disease (123). In addition, IHC in CRPC is typically only conducted to confirm the presence or absence of a particular splice variant (SV), such as AR-V7 in addition to AR-FL (122). To better address the issue of how prevalent AR-SVs are in prostate cancer and their prognostic and diagnostic value, we need to develop more precise and accurate quantification AR-SV proteins.

### Biophysical/biochemical approaches to quantitate AR-SVs

**Figure 5:**
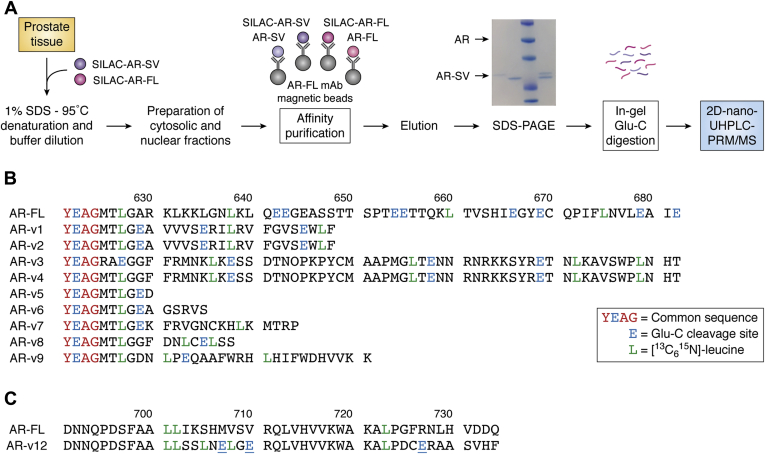
***A*, a work flow for SID-LC-IP-HRMS quantitation of AR variants.** The biospecimen is spiked with a SILAC-internal standard for an AR variant, the mixture is subjected to denaturation and immunoaffinity purified using Ab-magnetic beads. Eluents are subjected to SDS-PAGE and in-gel digestion with Glu-C. The unlabeled (biospecimen) peptides and labeled (internal standard peptides) are subjected to 2D-nano-LC-parallel reaction monitoring/MS where they behave identically except for differences in mass, top. *B*, amino acid sequences of AR-FL and AR-V1-AR-V9 in the region 620 to 682. *C*, amino acid sequences of AR-FL and AR-V12 in the region 700 to 734.

Stable isotope dilution–immunoprecipitation–liquid chromatography–mass spectrometry (SID-IP-LC-MS) offers a new approach to quantifying protein biomarkers (124, 125). This methodology has made it possible to rigorously quantify circulating proteins in the plasma (126, 127), serum (127, 128), and circulating blood cells (129) with precision and accuracy typical of that attained for drugs and endogenous small molecules. SID-IP-LC-MS can also be used for the quantification of protein splice variants (126) as well as posttranslationally modified proteins (127) in the systemic circulation. Therefore, SID-IP-LC-MS-based methodology should be extremely useful for the quantification of the AR-FL and AR-SVs in tumor tissues and CTCs. In this method AR internal standards are generated by stable isotope labeling in cell culture (SILAC). The SILAC standard is added to the extract of interest and behaves identically to the unlabeled protein through immunoprecipitation, in-gel protease digestion and LC-HRMS (see Fig. 5, top). The ratio of unlabeled AR peptide: labeled AR peptide *versus* AR amount provides a calibration curve from which it is possible to precisely and accurately determine AR amount. Such an approach has never been applied to SHR. Such a method would allow the quantitation of AR-FL and AR-SV in different cellular compartments and whether the amount of AR-SV changes as CRPC advances.

IP-LC-HRMS has been employed in proteomic approaches to detect ERα and ERβ, and peptide coverage showed that the majority of ERβ antibodies did not detect the receptor (130, 131). Thus these methods are fundamental in measuring accurately and precisely SHR amounts in any tissue (130). Mass-spectrometric-based studies of the AR have been restricted to quantifying androgens in tumor tissue (132), confirming AR sequences (133), identifying AR phosphorylation sites (134), identifying AR-binding partners (135), and establishing evidence for AR dimerization (136). In contrast to RNA or antibody-based methods, MS-based methods can potentially quantify all 22 known AR-SVs identified in CRPC with high precision and accuracy to comprehensively understand their biological role in disease and to provide the basis for evaluation as clinical biomarkers.

The SID-IP-LC-MS method in combination with endoproteinase Glu-C (V8 protease) digestion (127) could be used in the future to specifically quantify AR-FL together with all the AR-SVs in tumor tissues and CTCs. Thus, total AR (AR-FL + AR-SVs) would be quantified using specific peptides (Fig. 5*B*, bottom panel). AR-FL would be quantified using the Glu-C AR-FL-specific peptide (A^623^GMTLGARKLKKLGNLKLQE^642^; Fig. 5*B*), with stable isotope labeling by amino acids in cell culture (SILAC)-A^623^GMT**L**GARK**L**KK**L**GN**L**K**L**QE^642^ derived from concomitant Glu-C digestion of SILAC-AR-FL as the internal standard (Fig. 5*B*; **L** = [^13^C_6_^15^ N]-leucine). Individual AR-SVs could be quantified from their specific Glu-C-peptides. For example, K^630^FRVGNCKHLKMTRP^644^ and R^711^QLVHVVKWAKALPDCE^727^ (Fig. 5, *B* and *C*) would be used to quantify AR-V7 and AR-V12, respectively, using appropriate SILAC-AR-SV protein internal standards.

## Feeding the AR with ligand

The preceding sections deal with AR. However, CRPC can evade the effects of ARSI therapy by synthesizing more ligand. AKR1C3 (type 5 17β-hydroxysteroid dehydrogenase) plays an essential role in intratumoral biosynthetic pathways to T and DHT, which are potent ligands for the AR. AKR1C3 is the most upregulated steroidogenic enzyme in CPRC (137). It is induced by androgen deprivation in prostate cancer cells, xenografts, and patient tumor samples (138). As a consequence, AKR1C3 is considered a biomarker for advanced prostate cancer (139, 140, 141). Based on eight different preclinical studies, it is estimated that AKR1C3 is overexpressed in at least one-third of CRPC patients (138, 139, 140, 141, 142, 143, 144, 145). AKR1C3 is induced by TMPRSS-ERG (a fusion protein that appears in advanced prostate cancer based on Gleason grade) (144). ERG acts as a transcription factor to induce AKR1C3, which in turn synthesizes androgens to increase TMPRSS-ERG expression, which can surmount the repressive effects of AR on the *AKR1C3* promoter. In support of this model ChIPseq experiments demonstrated recruitment of ERG to the *AKR1C3* promoter/enhancer (144).

AKR1C3 is also overexpressed in C4-2B (metastatic prostate cancer) cells that are resistant to enzalutamide and abiraterone, and this resistance can be surmounted by indomethacin, a potent and selective competitive inhibitor of the enzyme, *in vitro* and *in vivo* (146, 147, 148). This has led to the use of indomethacin in clinical trials in patients that have progressed on enzalutamide (NCT02935205) and abiraterone (NCT0254990). More recently, the NSAID activity of indomethacin was eliminated by medicinal chemical optimization while retaining the potent and selective competitive inhibition of AKR1C3 (149). While these new indomethacin analogs offer promise to surmount drug resistance to ARSI therapy, drug resistance could still emerge due to changes in AR itself or the appearance of AR-SVs

Nonetheless, AKR1C3 remains an attractive target since it has other unexpected functions that impact its role in CRPC. It stabilizes the Siah2 ubiquitin ligase preventing its self-ubiquitination and permits degradation of AR-NCoR complexes (150) and it stabilizes AR-V7 (151). Whether small-molecule inhibitors of AKR1C3 can block these important functions is unknown.

**Figure 6:**
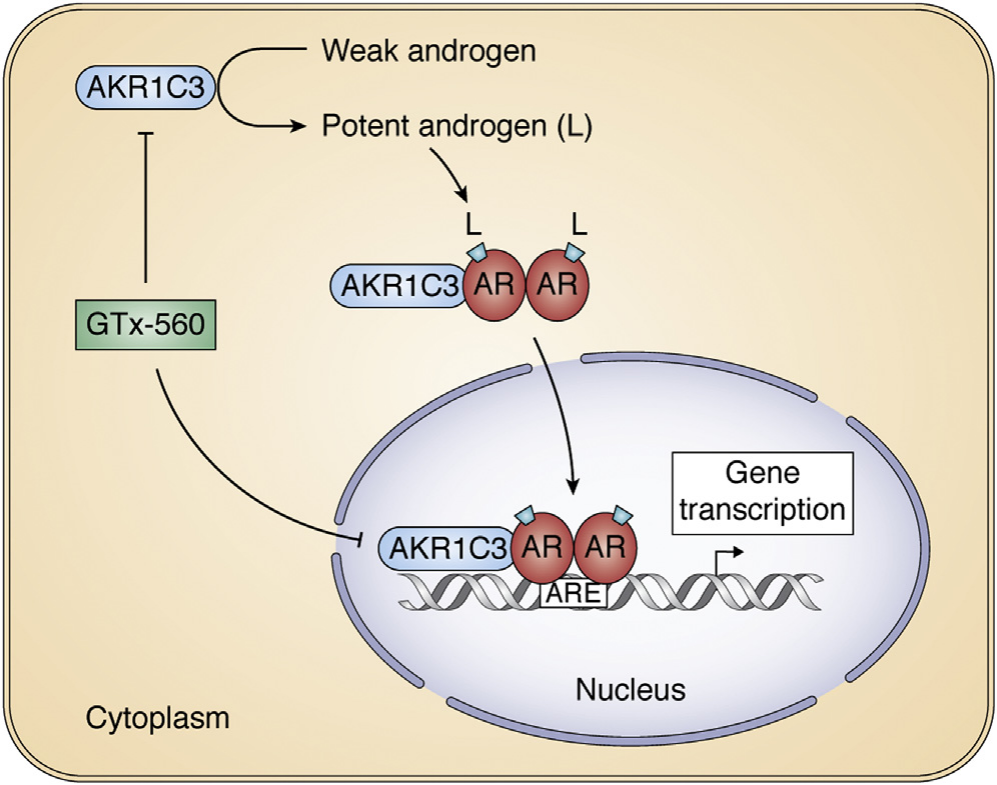
**AKR1C3 acts as a 17β-hydroxysteroid dehydrogenase and as a coactivator of the androgen receptor (AR).** AKR1C3 converts weak androgens (4-androstene-3,17-dione and 5α-androstane-3,17-dione) to potent androgens (T and DHT = L) and translocates to the nucleus with AR where it acts as a coactivator. Both the enzymatic function of AKR1C3 and its coactivator function are blocked by GTx-560.

AKR1C3 is also unique in that it is the only steroidogenic enzyme that acts as a coactivator of the AR (152). AKR1C3 is a selective coactivator of AR, having no effect on the transcription mediated by other SHRs (152). The coactivator function of AKR1C3 can be abolished by GTx-560 a competitive enzyme inhibitor suggesting that it is a druggable AR selective coactivator target (153), Figure 6. The coactivator domain of AKR1C3 maps to amino acid residues 171 to 237 that is distinct from its active-site, steroid-binding cavity and its cofactor-binding site (152). The highly related AKR1C1 and AKR1C2, which share 86% sequence identity, do not act as AR coactivators (152). Comparison of the 171 to 237 region in all three enzymes shows a difference in amino acid residues in these enzymes. In AKR1C3, the sequence corresponds to ^222^QRDKRW^227^, whereas in AKR1C1 and AKR1C2, the sequence corresponds to ^222^HREEPW^227^. Mapping this region to the AKR1C3 crystal structure shows that this peptide is in a disordered loop. Although p160 coactivators contain an LxxLL motif that binds to the LBD (154, 155), p160 coactivators also increase transcription by activating the NTD of AR in a ligand-independent manner. In this instance, the LxxLL motif is not required and instead a “Q” rich region of p160 binds to the Tau-5 domain of AR (62), Figure 2. It is this crucial interaction between the NTD and AKR1C3 that is most likely responsible for the coactivation of AR-FL. Mapping of the coactivator region could be accomplished by additional mutagenesis on AKR1C3 and reverse mutagenesis on AR-FL. The value of this approach would be to map the peptides responsible for the protein–protein interaction (PPI), so that PPI inhibitors based on peptidomimetics could be developed to block this interaction.

Physical interaction of AKR1C3 and AR-FL receptor has been demonstrated by co-IP and proximity ligation assays. Confocal microscopy shows that AKR1C3 cotranslocates with the AR to the nucleus in transfected HEK-293 cells (152). Whether AKR1C3 alters the transcriptome mediated by AR-FL in the presence of ligand remains to be determined. It is also unknown whether AKR1C3 can act as chaperone for unliganded AR-FL and promote its translocation to the nucleus. Whether AKR1C3 can act as coactivator of the AR-SVs and alter their transcriptomes in an AR-variant-dependent manner remains to be determined.

### Biophysical approaches—AKR1C3 structure-function

A large number of monofunctional competitive inhibitors based on different structural scaffolds have been developed for AKR1C3 (156), and there are many AKR1C3•NADP^+^•Inhibitor complexes available in the PDB (149, 157, 158). However, the crystal structure of AKR1C3•NADP^+^•GTx-560 has not been determined. Knowledge of this structure could lead to the development of superior bifunctional agents to GTx-560 that are AKR1C3-specific. GTx-560 is equipotent as an AKR1C1 and AKR1C2 competitive inhibitor, and thus it is an important probe to elucidate how it acts allosterically through the AKR1C3 structure to eliminate coactivator function, but it cannot be developed as a drug. It is unlikely that a crystal structure will be able to provide information on the dynamic perturbations that permit GTx-560 to act as a competitive enzyme inhibitor and block AKR1C3 coactivator function *via* a long range (allosteric effect) on the disordered loop. Recent advances in solution NMR of proteins will provide detailed characterization of the structural and dynamic perturbations responsible for the bifunctionality of GTx-560. As a companion to structural techniques such as crystallography and cryo-EM, solution NMR provides access to otherwise hidden attributes such as transient populations of critical minor states (159) or dynamic manifestation of conformational entropy (160, 161) to understanding fundamental biophysical events such as ligand binding and its allosteric long-range effects.

AKR1C3 represents a significant challenge to both solution NMR and drug discovery and development. Recent advances in preparation of the 37 kDa protein with appropriate isotopic labeling avoid the need to refold the protein, which is often a debilitating issue for proteins of this size and structural complexity (162). This sets the stage for examination of the structural, dynamical, and thermodynamic basis for examining how bifunctional inhibitors such as GTx-560 bind and thereby illuminate potential paths to more potent bifunctional agents. Furthermore, NMR can be particularly useful in the context of drug discovery by using reverse-micelle encapsulation of AKR1C3. The confined space effect enables cost-effective screens of low-affinity binders, such as those anticipated in fragment libraries that escape detection by activity-based assays or other biophysical techniques (163). The greater flexibility in detection and elaboration of “hits” discovery through the application of FBDD using RM-NMR will provide ways to differentiate AKR1C3 from its close homolog AKR1C2, which inactivates DHT (164). Such agents offer the promise to eliminate AR ligand synthesis in CRPC and also block the transactivation of the AR-FL enabled by the coactivator function of AKR1C3 without affecting AKR1C2. Use of FBDD as described by Fesik and colleagues has led to the identification of more than 30 drug candidates (165, 166). The same technology could be used in peptide scanning of fragments of the AR-NTD in encapsulated AKR1C3 to define structural perturbations that will disclose its coactivator domain that may not be easily identified by site-directed mutagenesis.

## Summary

Progress to eradicate the AR axis in CRPC involves stopping the AR in its tracks. Progress to this objective could be achieved by elucidating the structure of AR variants and their complexes by cryo-EM. However, an ensemble of structures may exist due to the intrinsically disordered NTD and its stabilization by ligand and binding partners to obtain a single high-resolution structure may not be possible.

The intrinsic disorder of the NTD may be key to AR function, and studies on its structure and interdomain coupling will be important for the rational design of NTD antagonists and PPI inhibitors to block coregulator function. These potential therapeutics would target AR even when the LBD is absent and would be particularly attractive to block signaling through AR-SVs. An alternative strategy to eliminate AR signaling has been the use of Proteolysis Targeting Chimeras (PROTAC). However, the PROTAC derivative based on enzalutamide, *e.g.*, ACC-4, will be ineffective in degrading AR-SV, which lacks the LBD (167).

As the AR does not act alone, targeting specific coregulators that interact with AR to restore its activity in ARSI-resistant tumors offers a promising approach and could involve targeting MED1 with CDK7 inhibitors. Several CDK7 inhibitors are in clinical trials for solid tumors, but only one trial is enrolling CRPC patients so that the efficacy of this approach remains to be determined.

The AR uses a number or escape mechanisms to evade the benefit of ARSI therapies. This includes the appearance of AR-SVs. AR-SVs may well be important biomarkers of disease progression and response to ARSI therapy but without a reliable assay to measure protein mass, these biomarkers cannot be assessed in terms of their sensitivity and specificity or clinical utility. If this were achieved using mass spectrometry, then the appearance of AR-SVs could be used to predict lack of response to ARSI therapy and promote the use of other therapies such as taxanes in their place. Another escape mechanism involves increased AR ligand synthesis catalyzed by AKR1C3. AKR1C3 is not only involved in ligand synthesis but AR coactivation as well. Bifunctional agents based on GTx-560, which target both functions of AKR1C3, would be an approach provided the protein is confirmed as a major AR coactivator in CRPC.

CRPC is a heterogenous disease and that different phenotypes can exist. For example, small-cell neuroendocrine prostate cancer can emerge after ARSI therapy in about 20% of patients that progress (168). Diverse drivers of disease progression have also been identified by characterizing CRPC metastases and patient-derived xenografts. For example, a double-negative phenotype that is AR and neuroendocrine gene null has been identified that may remain refractory to current therapies or approaches (169). Nonetheless, progress toward eradicating AR signaling in CRPC can be made using the approaches described in this review and could benefit patients with this disease.

## Conflict of interest

T. M. P. is the founder of Penzymes, LLC; he receives sponsored research funding from Forendo and serves on the Expert Panel for the Research Institute for Fragrance Materials. I. A. B is a founder of Proteoform Bio and a paid consultant for Calico, Chimerix, PTC Therapeutics, Takeda Pharmaceuticals, and Vivo Capital. S. R. P is the president of ProsTech, Inc. All other authors declare no competing interests.
